# Advanced Photocatalytic Degradation of Cytarabine from Pharmaceutical Wastewaters

**DOI:** 10.3390/toxics12060405

**Published:** 2024-05-31

**Authors:** Alexandra Berbentea, Mihaela Ciopec, Narcis Duteanu, Adina Negrea, Petru Negrea, Nicoleta Sorina Nemeş, Bogdan Pascu, Paula Svera (m. Ianasi), Cătălin Ianăşi, Daniel Marius Duda Seiman, Delia Muntean, Estera Boeriu

**Affiliations:** 1 Faculty of Industrial Chemistry and Environmental Engineering, Politehnica University Timişoara, Victoriei Square, no. 2, 300006 Timisoara, Romaniaadina.negrea@upt.ro (A.N.); petru.negrea@upt.ro (P.N.); 2 Research Institute for Renewable Energies—ICER, Politehnica University Timisoara, Gavril Musicescu Street, no. 138, 300774 Timisoara, Romania; ioan.pascu@upt.ro; 3 National Institute for Research and Development in Electrochemistry and Condensed Matter, 144th Dr. A. P. Podeanu Street, 300569 Timisoara, Romania; 4 Coriolan Drăgulescu’ Institute of Chemistry, Bv. Mihai Viteazul, No. 24, 300223 Timisoara, Romania; 5 Department of Cardiology, Victor Babes University of Medicine and Pharmacy Timisoara, 2 Piata Eftimie Murgu, 300041 Timisoara, Romania; daniel.duda-seiman@umft.ro; 6 Multidisciplinary Research Centre on Antimicrobial Resistance, Department of Microbiology, “Victor Babes” University of Medicine and Pharmacy, 2 Eftimie Murgu Square, 300041 Timisoara, Romania; 7 Department of Pediatrics, Victor Babes University of Medicine and Pharmacy Timisoara, Eftimie Murgu Square 2, 300041 Timisoara, Romania; estera.boeriu@umft.ro

**Keywords:** cytarabine Kabi, degradation, UV radiation, kinetic process

## Abstract

The need to develop advanced wastewater treatment techniques and their use has become a priority, the main goal being the efficient removal of pollutants, especially those of organic origin. This study presents the photo-degradation of a pharmaceutical wastewater containing Kabi cytarabine, using ultraviolet (UV) radiation, and a synthesized catalyst, a composite based on bismuth and iron oxides (BFO). The size of the bandgap was determined by UV spectroscopy, having a value of 2.27 eV. The specific surface was determined using the BET method, having a value of 0.7 m^2^ g^−1^. The material studied for the photo-degradation of cytarabine presents a remarkable photo-degradation efficiency of 97.9% for an initial concentration 0f 10 mg/L cytarabine Kabi when 0.15 g of material was used, during 120 min of interaction with UV radiation at 3 cm from the irradiation source. The material withstands five photo-degradation cycles with good results. At the same time, through this study, it was possible to establish that pyrimidine derivatives could be able to combat infections caused by *Escherichia coli* and *Candida parapsilosis*.

## 1. Introduction

The removal of pharmaceutical compounds (medicines, anticancer agents, diagnostic agents, cosmetics) from wastewater represents a major challenge in the actual context of the development of human society. This is because most of these compounds, such as anticancer agents, cannot be removed from water through biological degradation, therefore remaining in the environment for a long time [1,2]. Anticancer agents are designed to disrupt or prevent cell proliferation, usually by interfering with DNA synthesis, indicating strong cytotoxic, genotoxic, and mutagenic effects in several organisms [3].

Although the concentrations of these substances in the environment are generally lower than those of other classes of drugs, concerns have arisen regarding the impact of anticancer agents on the environment [4], including the microbial flora in waters and soils where these agents can accidentally end up.

One of the most used anticancer agents is Kabi cytarabine [4]. Cytarabine belongs to the antimetabolic group of drugs [5], which interacts directly with human DNA [6] and is used in chemotherapy to treat ovarian cancer [7,8], acute myeloid leukemia [9,10,11], lymphoblastic leukemia [12,13], and non-Hodgkin lymphoma [4,14,15]. The mechanism of action of cytarabine can be described as follows: after its penetration into cells, cytarabine is transformed into cytarabine-5′-triphosphate, which is its active metabolite. Subsequently, the metabolite competes with cytidine to incorporate it into developing DNA [13,16]. The DNA repair and replication process is inhibited, especially during the S phase of the cell cycle, making it a specific drug for rapidly dividing cells, such as cancer cells [5,13]. The effects are not only manifested in cancer cells, but the adverse effects can also reach throughout the body, including leukopenia, thrombocytopenia, anemia, fever, anorexia, nausea, vomiting and aseptic meningitis [16,17]; therefore, a process of oxidative degradation of such compounds is necessary.

A classic process of oxidative degradation of cytarabine is the Fenton process, which involves the generation of hydroxyl (${\mathrm{OH}}^{*}$) and hydroperoxyl (${\mathrm{OOH}}^{*}$) radicals through the redox decomposition process of H_2_O_2_ in the presence of Fe^2+^ ions, acting as a catalyst in an acidic environment (pH ~ 3) [18,19]. The mechanism of radical generation is presented below [18,20]:

(1) $$H_{2}O_{2\;}+{\mathrm{Fe}}^{2+}\to {\mathrm{Fe}}^{3+}+{\mathrm{OH}}^{-}+{\mathrm{OH}}^{*}$$

(2) $$H_{2}O_{2\;}+{\mathrm{Fe}}^{3+}\to {\mathrm{Fe}}^{2+}+H^{+}+{\mathrm{OOH}}^{*}$$

These are the main reactions involved in the decomposition of H_2_O_2_, but the process is much more complicated, involving other secondary reactions, such as radicals with an auto-scavenging tendency and the generation of organic radicals [21,22]. Although the Fenton oxidation process is a high-performance process, it presents a multitude of disadvantages, such as a need for pH adjustment [23], high costs due to the addition of H_2_O_2_ [24], and a large amount of sludge with iron content is produced [23], coupled as well with the H_2_O_2_ degradation in the case of its storage. Due to these inconveniences, new methods of oxidative degradation of organic pollutants, such as photocatalytic ones, were desired.

**Figure 1:**
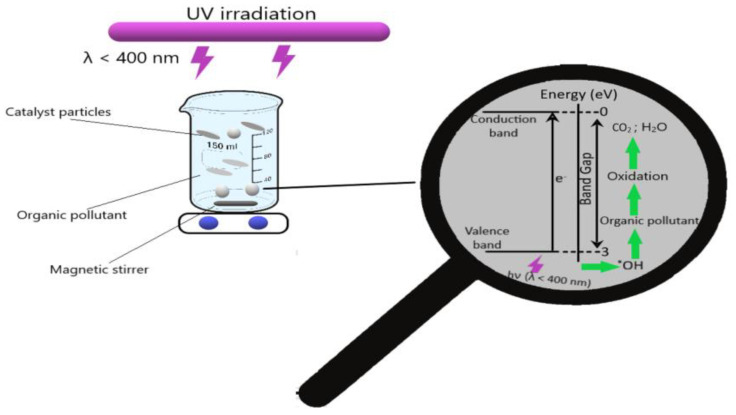
Photocatalysis process.

Photocatalysis offers a variety of advantages: easy operation, high efficiency, low energy consumption, and minimal secondary compound generation [25,26]. This method is based on the use of semiconductor particle suspension irradiated with UV light. UV irradiation leads to excitation of electrons from the valence band and their displacement to the conduction band, resulting in the in situ generation of strong oxidizing agents, such as hydroxyl free radicals (•OH) [27]. Schematically, the photocatalytic degradation is represented in Figure 1.

To try to make the photocatalysis process more efficient, various types of materials were synthesized: gold nanoparticles (AuNPs) [28], titanium dioxide (TiO_2_) [29,30,31,32], silver phosphate (Ag_3_PO_4_), silver vanadate (AgVO_3_), silver carbonate (Ag_2_CO_3_), silver/titanium dioxide (Ag/TiO_2_), silver/zinc oxide (Ag/ZnO), chitosan/silver (CS/Ag), reduced graphene oxide/silver nanoparticles (r-GO/Ag), black iron oxide/silver phosphate and tungsten oxide (Fe_3_O_4_/Ag_3_PO_4_@WO_3_) or silver/silver oxide (Ag-Ag_2_O) [33,34,35,36,37]. However, all of these catalysts present a multitude of disadvantages: low availability and high costs; low removal capacity of hydrophobic pollutants, irregular dispersion in aqueous suspension, as well as the need for post-treatment recovery of the catalyst [38].

In this context, in the present work, the obtaining of a composite material based on iron and bismuth, a material that would present photochemical properties useful for the degradation of cytarabine, was studied.

Composite materials based on iron and bismuth are of particular interest, primarily due to the abundance of iron [39], and secondly, iron oxide (Fe_2_O_3_) is chemically stable in aqueous media, having a small band gap and being economically advantageous [40]. Bismuth-based compounds represent an attractive solution due to the small width of the band gap and high stability [41], as well as their ability to degrade a wide range of pollutants: inorganic dyes [42], volatile organic compounds [43], pharmaceutical compounds [44], as well as nitrogen oxides [45].

Considering all the above mentioned, an alternative to improve the Fenton process is its combination with UV light, which in this way will achieve the homolytic breakdown of H_2_O_2_, increasing the amount of •OH, this being the main purpose of the Fenton method. From a stoichiometric point of view, two •OH radicals should be generated from one molecule of H_2_O_2_, but due to auto-scavenging it is necessary to operate with larger amounts of H_2_O_2_. The most important role of UV irradiation in combination with the Fenton method is the generation of Fe^2+^ ions. UV–Vis radiation, especially below 450 nm, is capable of transferring excited Fe^3+^ charges, dissociating them into Fe^2+^ and an oxidized ligand, which can be any Lewis base capable of complexing with the ferric ion. In some cases, these oxidized ligands can generate •OH radicals after UV irradiation. Furthermore, Fe^3+^ ions are, as a result, photo-reduced, leading to a significant decrease in the speed of the Fe^2+^ ions’ regeneration process, and at the same time, a significant drop in sludge residue with iron content has been observed.

It should also be mentioned that, in this type of heterogeneous system (Fenton-UV), UV radiation plays a crucial role, especially in combination with minerals that contain iron and are slightly photoactive, or even in the case of iron deposited on a substrate of an active carbon catalyst, due to the excitation of delocalized electrons in the carbon matrix [18,19]. The Fenton process mechanism combined with UV light is presented below [18,19]:

(3) $${\left[\mathrm{Fe}\left(\mathrm{OH}\right)\right]}^{2+}+h\nu \to {\mathrm{Fe}}^{2+}+\mathrm{HO}•$$

(4) $${\left[\mathrm{Fe}(H_{2}O)\right]}^{3+}+h\nu \to {\mathrm{Fe}}^{2+}+\mathrm{HO}•+H^{+}$$

There are studies that follow the degradation of phenols by the Fenton method under UV irradiation, and that were successful both in the presence and in the absence of H_2_O_2_. It indicates, however, that these results occurred due to the adsorptive properties of the Fe/Active Carbon type material used for this purpose [46].

Therefore, this research paper presents the synthesis of a chemically stable material with high photocatalytic efficiency, further used to achieve the efficient degradation of cytarabine. The material is a composite based on bismuth and iron oxides, Bi_x_Fe_1-x_O_y_. It should be mentioned that the cytarabine degradation process occurred in the absence of H_2_O_2_, or another solution with the role of an oxidative agent, a fact that brings novelty to this work.

At the same time, Kabi cytarabine antimicrobial activity was also investigated, starting from the consideration that this compound is used to stop cell proliferation, as claimed in the description of the therapeutic effect of the drug. To determine if cytarabine is able to stop the human cancer cell proliferation, we also tried to highlight its behavior on bacterial cells. In both situations, the selective toxicity of the drug [47,48,49,50] is essential in order to obtain the desired therapeutic effect. Such a desired therapeutic effect means inhibiting cancer cells and bacteria that can cause infections that would complicate the health of cancer patients. Based on that, in medical practice is desirable to have both effects: direct therapeutic effect (inhibition of cancer cells) and a cumulative concurrent effect (antimicrobial effect—especially to limit antibiotic resistance). Thus, drugs used in chemotherapy should be effective or even slightly toxic to some microorganisms, even at low concentrations.

Therefore, it is extremely important to know whether these therapeutic agents, in the present case Kabi cytarabine, can affect the oral or gastrointestinal microbiota, because it could cause the worsening of the condition of patients with oncological conditions [51,52] for which cytarabine is primarily used.

In the present work, the process of the degradation of a specific drug used in the field of oncology (Cytarabine Kabi) was investigated. It is well known that the process of drug elimination from the human body is urinary excretion, hence the possibility of its presence in urban sewage. That is why the present paper’s research is related to the treatment of wastewaters loaded with organic compounds; in this case the target compound being cytarabine. In general, drugs that end up in wastewater pose many problems in wastewater treatment because they are chemically stable, have a long retention time and are not easily degraded [53,54,55]. In general, the wastewater treatment plants use the traditional treatment process (involving mechanical, chemical and biological steps), paying no attention to the different organic pollutants that reach treatment plants and which pose a high danger to the biological flora needed to achieve the biological treatment of wastewaters. On the other hand, the long-term use of different drugs can increase bacterial resistance. This is why the antibacterial activity of cytarabine Kaby was evaluated on some microorganisms that under normal conditions are not pathogenic factors, able to be present in the human body without infection manifestations (*Staphylococcus* spp., *Candida* spp.), and on some bacterial strains known to present bacterial resistance (*Pseudomonas* spp.).

## 2. Materials and Methods

### 2.1. Synthesis and Characterization of BFO Material

**Figure 2:**
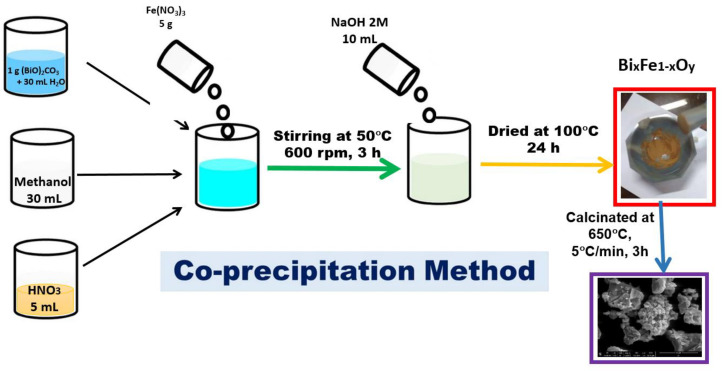
Synthesis of the BFO composite material.

To obtain the BFO composite, the co-precipitation method was used. In a suspension obtained by adding 1 g of bismuth (III) precursor—basic carbonate (Merck, Sigma Aldrich, Darmstadt, Germany) in 30 mL of distilled water, 30 mL of methanol were added (Merck, Sigma Aldrich). The mixture was stirred for one hour, then 5 mL of HNO_3_ solution (Merck, Sigma Aldrich) with pH ~2 were added to the reaction mass, continuing to stir for another 30 min until (BiO)_2_CO_3_ has been dissolved. Further, 5 g of Fe(NO_3_)_3_ (Merck, Sigma Aldrich, Darmstadt, Germany) were added to the reaction mass, and then the temperature was increased to 50 °C. The obtained reaction mass was mixed for 3 h until complete homogenization. For precipitation to occur, 10 mL of NaOH solution (7.5 g of NaOH (Merck, Sigma Aldrich, Darmstadt, Germany) dissolved in 100 mL of distilled water) was quickly added to the reaction mass. The obtained precipitate was separated and further washed with excess distilled water. The final product was dried for 24 h at 100 °C and then calcined in Nabertherm LHT407GN furnaces. The calcination process took place at 650 °C, for 3 h in air. The synthesis of the material is presented schematically in Figure 2.

### 2.2. Thermogravimetric Analysis, DTG

Differential thermal analysis (DTG) was performed on the new prepared material in order to highlight the temperature dependence of the physical properties, by using a TGA/SDTA 851-LF Mettler-Toledo system. Thermal decomposition was carried out in the presence of air and the sample was heat treated in the range of 25–800 °C.

### 2.3. Fourier Transform Infra-Red Spectroscopy, FT-IR

The new prepared material was also characterized by recording the FT-IS spectrum by using a JASCO FT/IR-4200 apparatus (SpectraLab, Shimadzu, Kyoto, Japan).

### 2.4. X-ray Diffraction Analysis, XRD

XRD analysis was performed by recording the XRD spectrum using a D8 Advance-Bruker AXS system, with Mo-Kα radiation (αMo = 0.7093 Å). This analysis was performed to obtain information regarding the degree of crystallinity of the material, but also to identify the presence of multiple phases in the new prepared material.

### 2.5. Scanning Electron Microscopy (SEM)

Further, the prepared material was characterized by scanning electron microscopy, using a Quanta FEG 250 instrument (FEI, Hillsboro, OR, USA).

### 2.6. Atomic Force Microscopy (AFM)

The new prepared material and the thermally treated one were analyzed by atomic force microscopy (AFM). AFM images were obtained by Scanning Probe Microscopy Platform (MultiView-2000 system, Nanonics Imaging Ltd., Jerusalem, Israel) using only the intermittent mode in normal conditions (298 K). The analysis used a chromium-doped tip with a 20 nm radius and 30–40 kHz resonance.

### 2.7. Determination of Band Gap Size by UV Spectroscopy

To determine the size of the band gap, UV–Vis spectra were recorded in the 300–1000 nm range, using the Varian Carry 50 spectrophotometer (Agilent Technologies, Palo Alto, CA, USA). The most used model for diffuse reflection is the one proposed by Kubelka and Munk.

The Kubelka–Munk (K–M) model has a particularly simple solution in the case of semi-infinite samples [56]. All the geometric peculiarities of an inhomogeneous sample are condensed into one parameter, the scattering coefficient, s. The diffuse reflectance $R_{\infty }$ is given by the relation:

(5) $$R_{\infty }=1+\frac{k}{s}-\sqrt{\frac{k}{s}(2+\frac{k}{s})}$$

where k is the absorption coefficient of the sample (k = 4π k/λ); λ is the wavelength.

This relatively simple form is easily solved for $\frac{k}{s}$ yielding the familiar K–M transform:

(6) $$\frac{k}{s}=\frac{(1-{R_{\infty })}^{2}}{2R_{\infty }}$$

The K–M transform of the measured spectroscopic observable is approximately proportional to the absorption coefficient and hence is approximately proportional to the concentration. The internal scattering processes are taken into account by introducing the semi-empirical scattering coefficient into the theoretical description of the diffusive reflection. The internal scattering process is determined by the size of the particles and the refractive index of the sample, presenting a low dependence against the adsorption coefficient and the radiation wavelength. In this context, the K–M model considers the scattering coefficient constant. Experimental data proved that the scattering coefficient varies slowly with the variation of the wavelength. However, the most important variation is related to the packing density, so special attention must be taken for powder packing if quantitative results are required [55,56,57].

### 2.8. Determination of the Specific Surface Area

Additionally, the new prepared material was characterized by determining its specific surface by using the Brunauer–Emmett–Teller (BET) method, with a Quantachrome Nova 1200e instrument (Anton Paar GmbH, Osfildern-Scharnhausen, Germany).

### 2.9. Photochemical Degradation of Cytarabine

**Figure 3:**
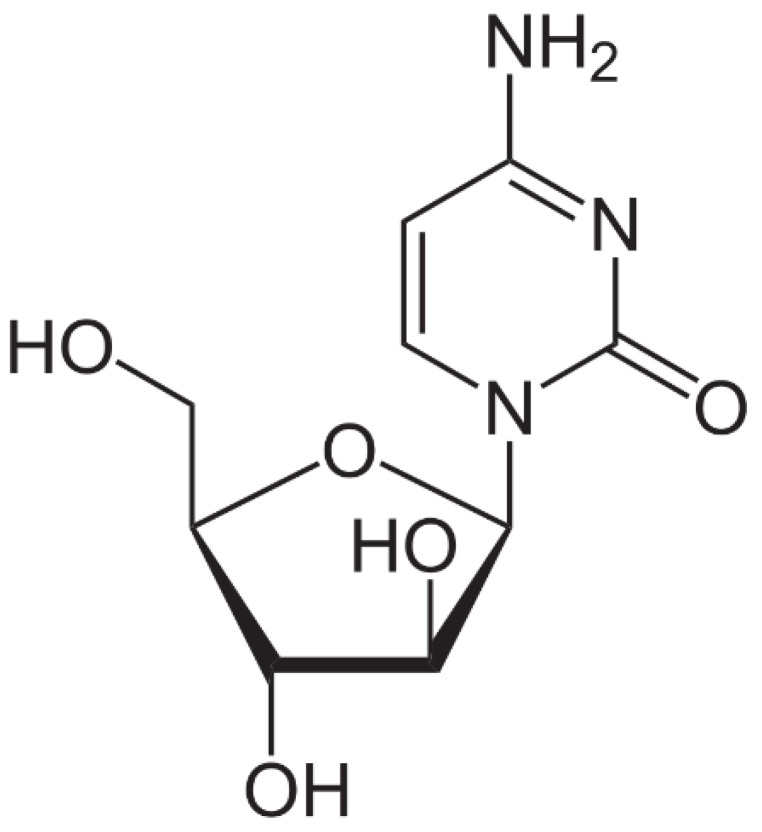
Cytarabine chemical structure [60].

Cytarabine is used in the form of an injectable or infusion solution, being clear, colorless, and having a pH between 7.0 and 9.5. This specific anticancer drug has a molecular mass of 243.086 uam and its chemical formula is presented in Figure 3. Cytarabine has a low biological stability, with the molecule undergoing a rapid deamination of the NH_2_ group at the level of the intestinal tract [58], and the carbohydrate part of the molecule preventing its rotation once it enters into the DNA [59]. Fresenius Kabi Oncology PLC manufactures this specific drug.

### 2.10. The Influence of Irradiation Time

Initially, cytarabine was placed in contact with the BFO material under stirring in the dark to determine if the new designed material exhibits adsorbent properties, by mixing 0.15 g of BFO with 50 mL solution with an initial concentration of 10 mg L^−1^ cytarabine. After 30 min the system was subjected to UV radiation from a distance of 3 cm.

**Figure 4:**
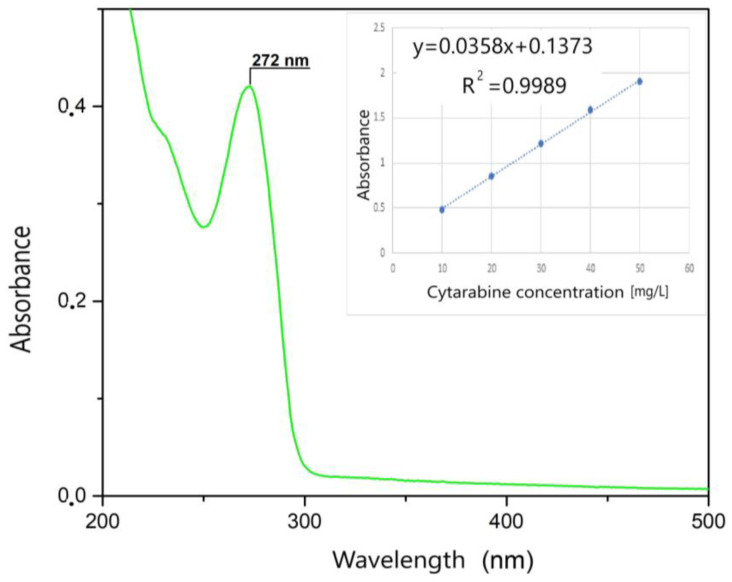
UV spectrum and the calibration line.

First, the UV spectrum was recorded in the 200–500 nm range only for the Kabi cytarabine solution (Figure 4). As can be seen, a specific peak for cytarabine appears at 272 nm, and this peak is specific to the group that is destroyed following the photochemical oxidation process. Then, the spectra were recorded for different initial concentrations of cytarabine. The calibration line was made by graphical representation of absorption = f (c), at the wavelength λ = 272 nm (Figure 4 inset). This calibration line was obtained in order to be able to determine the residual concentration of cytarabine.

### 2.11. The Influence of the Distance between the Irradiation Source and the Sample

To determine the optimal irradiation distance between the irradiation source and the sample, UV–Vis spectra of the samples exposed at the irradiation sources placed at different distances were recorded. Each sample, before UV irradiation, was left to adsorb in the dark for 30 min. The samples containing 50 mL of 10 mg/L cytarabine solution and 0.15 g of BFO material were then irradiated by placing the irradiation source at different distances (3, 7 and 10 cm). After an irradiation time of 120 min the UV spectra were recorded.

### 2.12. The Influence of the Amount of BFO Material

To determine the optimal amount of material required for the photocatalysis process, the amount of BFO material used was varied between 0.05 and 0.25 g using the same amount of cytarabine solution (50 mL of cytarabine solution with an initial concentration of 10 mg/L cytarabine). Each sample, before UV irradiation, was left to adsorb in the dark for 30 min, and the distance from the irradiation source was kept at 3 cm, for irradiation times in the range of 30–120 min.

### 2.13. The Influence of the Initial Concentration of Cytarabine

To establish the optimal concentration of cytarabine that can be degraded by the synthesized material, the initial concentration of cytarabine was varied in the range of 10 to 50 mg/L cytarabine (solutions containing 10, 15, 20, 25, 30, 35, 40, 45 and 50 mg/L were used). Each sample, before UV irradiation, was left to adsorb in the dark for 30 min. The distance of the sample from the irradiation source was 3 cm, the amount of material was 0.15 g, and the irradiation times varied in the range of 30–120 min.

The removal efficiency of cytarabine (R) was calculated by using the following equation:

(7) $$R=\frac{C_{i}-C_{t}}{C_{i}}\cdot 100\cdot [\%]$$

where $C_{i}$ and $C_{t}$ are the concentrations of cytarabine (mg/L) in the initial solution and after a time.

The photo-degradation process of cytarabine is described by the Langmuir–Hinshelwood equation, which can be simplified to a pseudo-first-order equation [61,62]:

(8) $$\ln\left(\frac{C_{0}}{C_{t}}\right)=kKt=k_{app}t$$

where *k* is the reaction rate constant (min^−1^);

*K* the adsorption coefficient of reactant (mg/L);

$k_{app}$ is the apparent rate constant (min^−1^);

and the slope of the plot $\ln\left(\frac{C_{0}}{C_{t}}\right)$ against time represents the apparent rate constant of cytarabine photo-degradation.

### 2.14. Photo-Degradation Cycles

The long-term stability of photocatalysts is a very important indicator for their practical application. To determine the photo-degradation cycles of the material, the considered system was irradiated for 120 min, and after each cycle, it was removed by filtration and then dried at 100 °C prior to the next usage of the photocatalyst.

### 2.15. Evaluation of the Antimicrobial Effect of Cytarabine

To evaluate the antimicrobial effect of cytarabine, microbial studies were carried out on bacterial Gram-negative strains (*Escherichia coli ATCC 25922, Pseudomonas aeruginosa ATCC 27853*) and bacterial Gram-positive strains (*Staphylococcus aureus ATCC 25923*). A reference fungal strain was also tested (*Candida parapsilosis ATCC 22019*). All microbial strains were provided by Microbiologics, Paris, France. During these tests a Mueller–Hinton (MH) culture medium (Sanimed International Impex, Bucharest, Romania) was used. The culture medium was prepared according to the manufacturer’s instructions, autoclaved, cooled to 45 °C, and poured into Petri dishes. The minimum inhibitory concentrations (MICs) of antimicrobial agents were determined by the agar dilution method, according to the European Committee for Antimicrobial Susceptibility Testing (EUCAST) standard [63].

The standardized inoculum (0.5 McFarland) was adjusted by dilution, obtaining a microbial suspension of 1–2 × 10^4^ CFU/mL. Subsequently, 1 µL of suspension was inoculated on MH agar, using a loop (Nuova Aptaca SRL, Canelli, Italy). After incubation at 35 ± 2 °C for 24 h, the MICs were calculated as the lowest concentration of cytarabine (μg/mL) that showed the complete inhibition of visible growth of test strains over a defined period (24 h in our study). Consequently, minimum bactericidal concentration (MBC) or minimum fungicidal concentration (MFC) was established as the lowest antimicrobial concentration (μg/mL) at which the entire concentration of the bacterial or fungal inoculum was reduced by 99.9%.

A positive growth control (medium with bacterial strain) was used, and the experiments were carried out in triplicate.

## 3. Results and Discussion

### 3.1. Physico-Chemical Characterization of BFO Material

**Figure 5:**
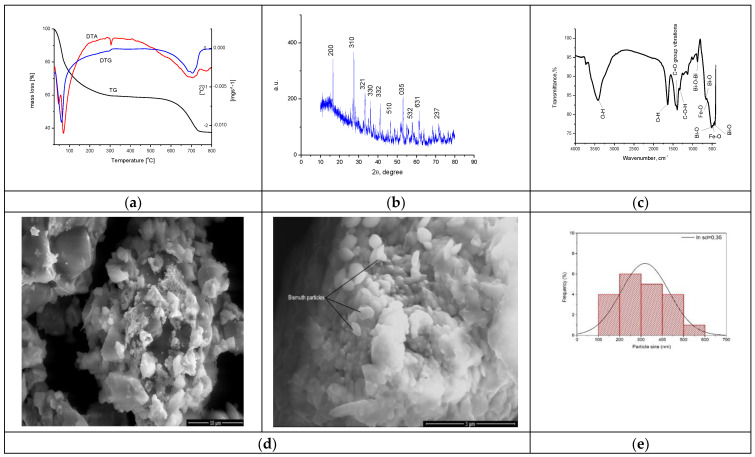

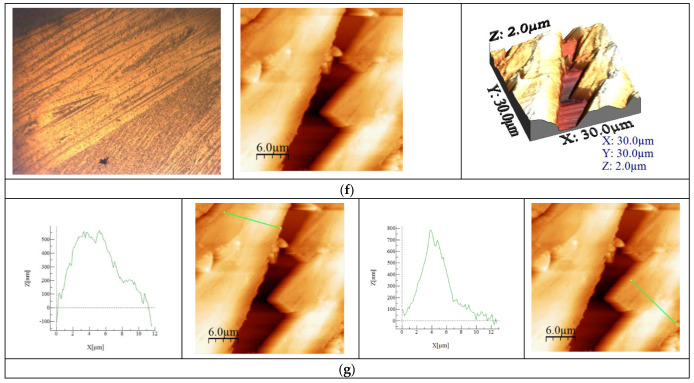
Physico-chemical characterization of BFO material. (**a**). TG, DTA, DTG curves for sample BFO; (**b**). X-ray diffraction pattern for BFO; (**c**). FT-IR Spectrum of BFO; (**d**). SEM analysis at 10,000×, 50,000×; (**e**). Particle distribution; (**f**). AFM images of BFO; (**g**). Calculated height for BFO on the selected areas.

The synthesized material was physico-chemically characterized by thermal analysis, X-ray diffraction, atomic force microscopy, infrared spectroscopy and by scanning electron microscopy, with the obtained information being depicted in Figure 5. The size of the band gap was determined by UV spectroscopy and the specific surface was investigated with the BET method.

The TG curve depicted in Figure 5a indicates a total mass loss of 62.39%. An analysis of the material highlighted the presence of three endothermic processes. In the first process, at 46 °C and 68 °C, losses of water and alcohol used in the system occur. In the second process, the decomposition of Fe(NO_3_)_3_ and (BiO)_2_CO_3_ is observed at 304 °C, corresponding to a mass loss of 41.29% [62]. In the last process, with a loss of 21.10%, the crystallization of bismuth oxide takes place through the total transformation of (BiO)_2_CO_3_, forming the BFO composite as the final product.

The XRD spectrum recorded for the synthesized BFO composite presented in Figure 5b was compared with the ICDD data sheet 01-082-1316 in order to confirm the synthesis of the desired BFO composite (Bi_25_FeO_40_). The crystallographic parameters indicate that a cubic system of type I23 was obtained [64]. The parameters of the unit cell a of the crystallographic structure show a value of 10.21 Å with a volume of the cell V = 1064.33 Å^3.^ In the crystallographic system obtained, it is observed that the Bi^3+^ ions occupy the octahedral positions and the Bi^5+^ and Fe^3+^ ions share the tetrahedral positions [65]. There are also situations where the Bi^3+^ ions occupy the tetrahedral position in the structure [66,67,68]. The mean size of the crystallites was calculated based on the Debye–Scherer equation, obtaining a value of 14.5 nm.

FT-IR analysis was performed to further characterize the groups found in the synthesized material, with the recorded spectrum being depicted in Figure 5c. At the wave number of 3416 cm^−1^, a vibration specific to the -O-H group appears [69], and such a specific vibration for -O-H group is also found at the wave number of 1635 cm^−1^ [70]. At the wave number of 1392 cm^−1^, a specific vibration associated with the presence of -C-O-H groups appears [69]. The bands located at wave numbers 1450 cm^−1^ and 1400 cm^−1^ are attributed to the stretching vibration of the C=O group [71]. At the wave number of 873 cm^−1^, a specific vibration associated with the Bi-O-Bi bound appear [69]. At the same time, at wave numbers 658 cm^−1^, 513 cm^−1^ and 421 cm^−1^, vibrations specific to Bi–O bonds appear [71].

Specific vibrations of the Fe-O group can be observed at the wave numbers of 508 cm^−1^ and 467 cm^−1^ [72,73,74,75]. Thus, the vibrations and bands observed in the recorded FT-IR spectrum confirm the preparation of the desired bismuth iron oxide material.

The SEM characterization provides essential information regarding the morphology of the material. SEM images for the obtained BFO composite are presented in Figure 5d. From the micrograph recorded at a magnification of 10,000, the formation of clusters with an uneven particle morphology is observed. Increasing the magnification to 50,000, we can see a slight arrangement of the particles in the shape of oval plates. According to the histogram in Figure 5e, the statistical representation of the particles indicates a size of 250 nm.

It is known that the specific surface plays an essential role in the reaction yield as well as in the adsorption yield of certain compounds [76]. Therefore, knowing the essential role of the specific surface of the material, we can determine the roughness of the material on the analyzed area using atomic force microscopy, which is in close correlation with the data obtained at BET.

Recorded microscopy images reveal the presence of an agglomerated needle formation in the case of the new prepared BFO composite. This difference becomes more evident when the AFM images are analyzed, revealing that during synthesis wires are created, with longer ones tending to overgrow smaller ones, as can be observed from the images depicted in Figure 5f. The data presented in Figure 5g show that the length of the wires have a value around 12 μm and a height between 500 and 700 nm. Based on the recorded AFM images, the values of specific parameters were calculated (Average roughness (Sa), Mean Square Root Roughness (Sq), Maximum peak height (Sp), Maximum valley depth (Sv), Maximum peak-to valley height (Sy)), the values of which are shown in Table 1.

Additionally, the heights of the particular surface formations were measured, providing the information regarding the uniformity of the clusters and/or particles. These measured heights can be directly compared with the maximum peak height (Sp) values, since the measurements were performed on the formations that presented higher heights (the lighter colored formations in the AFM images). In conclusion, the Sa value is positive but under three, meaning that the sample has low rugosity, which is also observed in the SEM images.

### 3.2. Band Gap Value Determination

**Figure 6:**
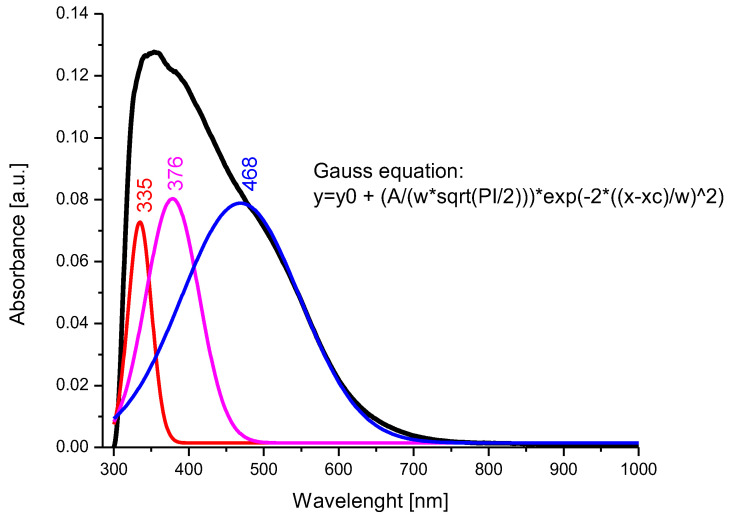
UV–Vis spectrum and deconvolution of BFO composite material.

In order to evaluate the value of the band gap the UV–Vis spectrum of BFO composite material was recorded, which is the spectrum depicted in Figure 6.

After the deconvolution of the recorded spectrum, the presence of three main bands located at 335 nm, 376 nm, and 468 nm was observed. The transitions of the bands from 376 nm to 468 nm, correlated with the data obtained by X-ray diffraction and FT-IR spectroscopy, indicate the occupation of the octahedral positions by the Bi^3+^ ions and the tetrahedral positions by the Fe^3+^ ions [76]. It is assumed that the band located at 335 nm can be attributed to Bi^3+^ ions, which occupy a tetrahedral position.

**Figure 7:**
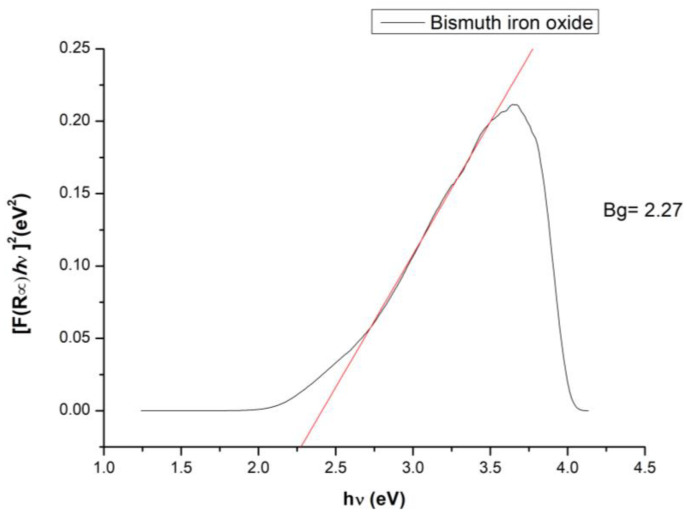
BFO band gap.

The band gap of the photocatalyst is an essential parameter for the photocatalytic capacity of the material, and generally a lower value of the band gap indicates an increased efficiency of the photocatalytic activity of the material [77]; therefore, from the UV–Vis spectrum obtained for the BFO material, the following band gap calculated with the Kubelka-Munk function appeared (Figure 7):

A band gap value of 2.27 eV indicates that the prepared BFO composite material is an ideal candidate for photocatalytic cytarabine degradation [78]. Different values obtained for the materials band gap are correlated with the different dimensions of the crystallites obtained during material synthesis. For example, a study performed by R. Koferstein et al. demonstrates that a BFO composite with a crystallite dimension around 75 nm presents a band gap of 2.7 eV, compared with the system having a crystallite dimensions of 15 nm which present a band gap of 2.2 eV [79].

### 3.3. N_2_ Sorption Isotherms

**Figure 8:**
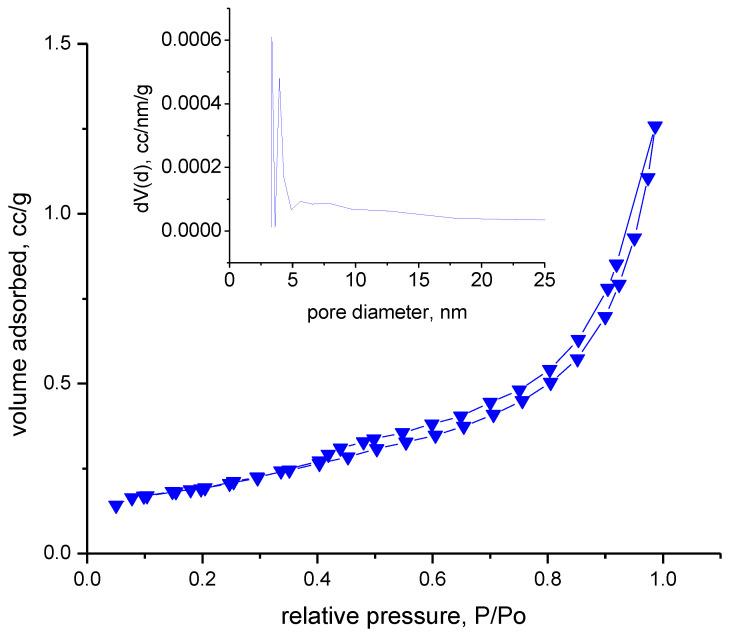
The adsorption–desorption isotherms.

The adsorption–desorption isotherms were recorded in a nitrogen atmosphere at 77 K. Prior to this, the samples were degassed in a vacuum at room temperature for 17 h. Analyzing the adsorption–desorption isotherms obtained, shown in Figure 8, we can specify that the material indicates type IVa with hysteresis type H3, specific to samples in the form of non-rigid aggregates with flat-like particles. In the inset figure presented in Figure 8, it can be seen that the pore distribution is multimodal, with most pores below 5 nm.

Based on the recorded adsorption/desorption isotherms, the textural parameters of the synthesized material, BFO, were calculated, which are presented in Table 2.

Evaluating the obtained data, it can be observed that the material is not porous, indicating a total pore volume of 0.002 cm^3^/g. The FHH method, which is corelated with AFM results, also indicates that the materials have low rugosity.

### 3.4. Studies on the Photochemical Degradation of Cytarabine

#### 3.4.1. Determination of the Optimal Irradiation Time

**Figure 9:**
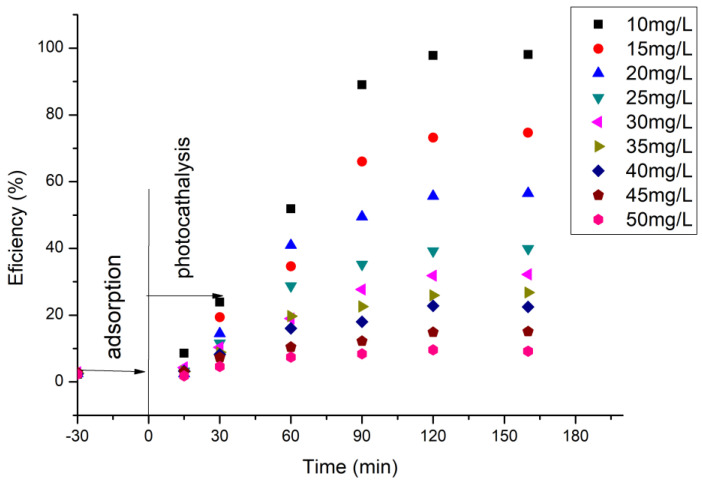
Dependence between cytarabine concentration and irradiation time.

To determine the optimal irradiation time, as well as the photocatalytic role of the BFO material, the UV–Vis spectra were recorded at different time periods, in order to determine the quantity of cytarabine oxidized based on the calibration line, and concomitant with the degradation process efficiency. The data obtained are depicted in Figure 9.

Based on the obtained experimental data, it can be established that, with the increase in UV irradiation time, 97.9% of the cytarabine in the solution is degraded after 120 min under UV radiation action. At the same time, it is observed that with an increase in the concentration of cytarabine, the efficiency of the material decreases in terms of the degradation of cytarabine, regardless of the UV exposure time. When an initial concentration of 50 mg/L cytarabine was used, after 30 min of dark adsorption and 160 min irradiation time, the removal of cytarabine was almost negligible (2.5%).

#### 3.4.2. Determining the Optimal Irradiation Distance

**Figure 10:**
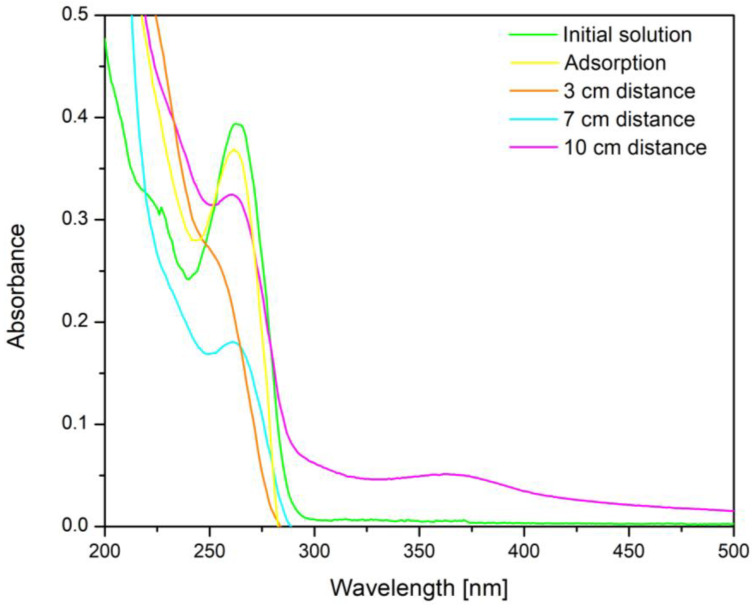
UV–vis spectra regarding the influence of the distance between the UV irradiation source and the BFO—cytarabine solution system.

To determine the optimal irradiation distance between the irradiation source and the sample, UV–vis spectra were recorded after the exposure to the BFO—cytarabine system (containing 10 mg/L cytarabine) for 160 min, with the UV source placed at different distances. The obtained spectra are presented in Figure 10.

From the analysis of the recorded UV–Vis spectra it can be seen that the distance between the UV irradiation source and the BFO—cytarabine solution system plays an important role. Thus, with the reduction in the distance between the UV irradiation source and the BFO—cytarabine solution system, the signal specific to cytarabine at 272 nm also decreases and implicitly disappears. This indicates that cytarabine is completely degraded at a distance of 3 cm between the UV irradiation source and the BFO—cytarabine solution system.

**Figure 11:**
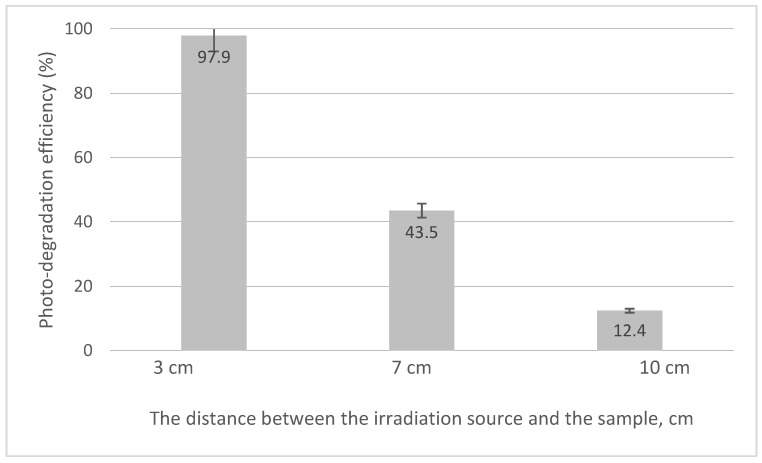
The distance between the irradiation source and the sample.

Based on the calibration curve, the values of photo-degradation efficiency obtained for different distances between UV source and the system were evaluated. The obtained data are presented in Figure 11.

From the experimental data, it can be stated that the distance between the irradiation source and the sample of 3 cm is optimal, leading at a maximum photo-degradation efficiency of 97.9%.

#### 3.4.3. Determination of the Optimal Irradiance and the Optimal Radiation Dosage

Calculating the optimal irradiance is necessary and essential, because it provides information about the optimal irradiation distance to facilitate the photocatalytic oxidation process of cytarabine. Therefore, to determine the optimal irradiance required for the photocatalytic degradation of cytarabine, Keitz’s formula was used [80]:

(9) $$P=\frac{I2\pi^{2}DL}{2\alpha +sin2\alpha }$$

From here we can find the irradiance, I:

(10) $$I=\frac{P(2\alpha +sin2\alpha )}{2\pi DL}$$

where P—the power of the ultraviolet lamp [W]

I—irradiation intensity [W m^−2^]

L—the length of the ultraviolet tube [m]

D—the distance travelled by the radiation to the sample [m]

$\alpha =\arctan[\frac{L}{2D}]$ rad; sin⁡α= $\frac{L}{\sqrt{4D^{2}+L^{2}}}$; $\cos\alpha =\frac{2D}{\sqrt{4D^{2}+L^{2}}}$; sin⁡2α=2 sin α cos α.

Based on this info, the following values are obtained (Table 3).

Following the conversion of the irradiation distance into irradiance, it turns out that the optimal irradiance is 250 W·m^−2^, because its absorbance indicates the lowest concentration of cytarabine present in the solution. Knowing the irradiance values, the optimal radiation dose can be determined (Table 4) using the optimal irradiance value of 250 W·m^−2^.

From the experimental results and UV–Vis analyses, it follows that the optimal radiation dose is 1,800,000 J/m^2^, or 1800 kJ/m^2^, which corresponds to the sample placed at a distance of 3 cm from the irradiation source. The optimum irradiation time is 120 min, with a material amount of 0.15 g, and a cytarabine concentration of 10 mg L^−1^ with a volume of 50 mL.

#### 3.4.4. Determination of the Amount of the Catalytic Material

**Figure 12:**
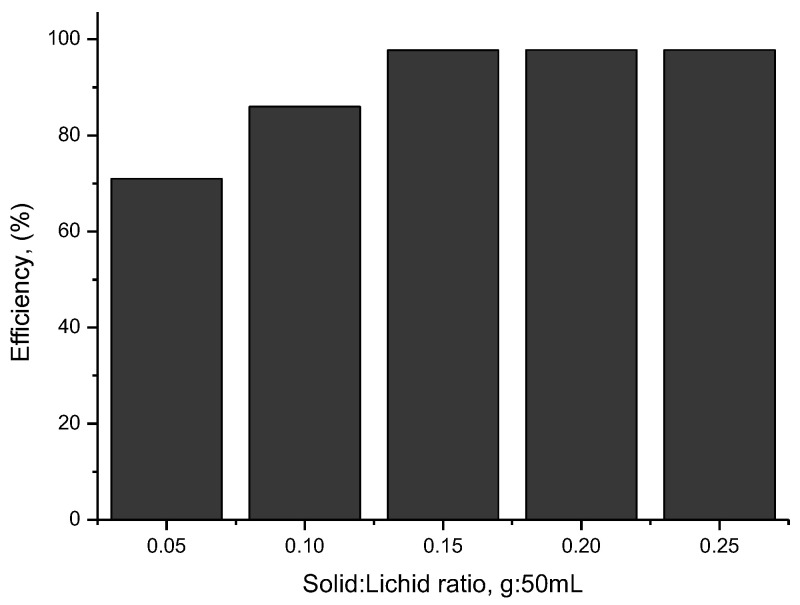
The influence of the amount of BFO material.

To determine the optimal amount of material required for the photocatalysis process, the amount of BFO material was varied in the range of 0.05–0.25 g, by using 50 mL of cytarabine solution which had an initial concentration of 10 mg/L (obtained data are presented in Figure 12).

From the data presented in Figure 12, it can be seen that 0.15 g of BFO material is the optimal amount required for the degradation of 50 mL cytarabine solution with an initial concentration of 10 mg/L. The optimal efficiency of the cytarabine degradation process in an aqueous medium is achieved when the S:L ration is 0.15 g:50 mL.

### 3.5. The Effect of the Initial Concentration of Cytarabine

**Figure 13:**
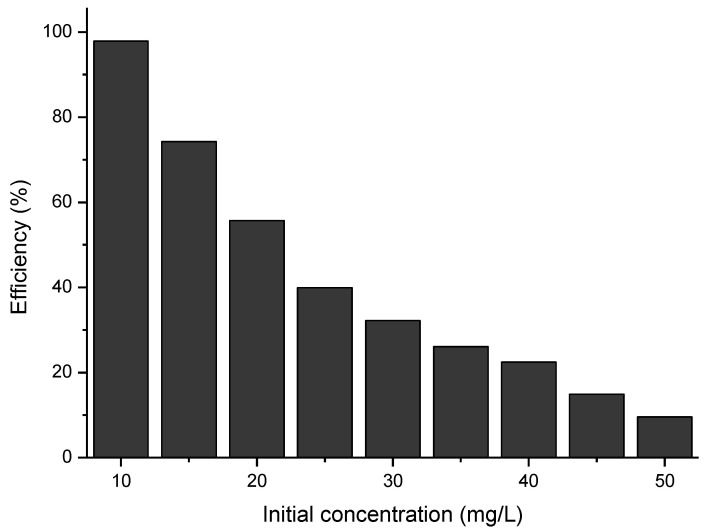
The influence of the initial concentration of cytarabine.

In order to follow the role of the initial concentration of cytarabine on the efficiency of the BFO material in terms of photo-degradation, the initial concentration of cytarabine was varied and the efficiency calculated. The obtained data are presented in Figure 13.

From the experimental data presented, for a ratio S:L = 0.15 g:50 mL, an irradiation time of 120 min, and a distance between the irradiation source and the sample of 3 cm, the maximum efficiency of 97.9% has been obtained for a cytarabine concentration of 10 mg/L.

### 3.6. Kinetic Studies

**Figure 14:**
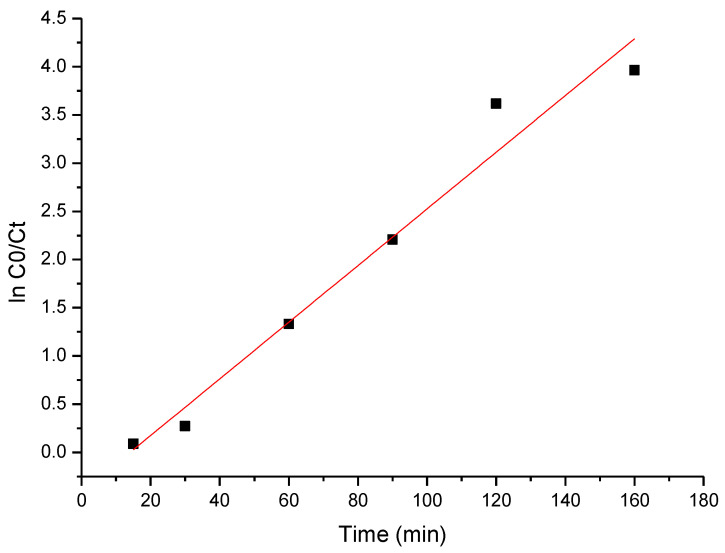
The pseudo-first-order kinetic plot of cytarabine degradation.

The obtained experimental data were modeled using the pseudo-first-order kinetic model, with the obtained results presented in Figure 14.

Based on the data presented in Figure 14, the specific parameters were calculated, which are listed in Table 5.

The obtained value of the apparent rate constant for cytarabine decomposition using BFO composites is 0.0307 min^−1^. The most important factors in expressing the photocatalytic activity are the phase content, crystallite size and specific surface area [81].

Undoubtedly, the improvement in photocatalytic efficiency is deeply related to the adsorption properties of the tested material. Adsorption is known to be one of the important steps in photocatalysis, because such a process takes place on the catalyst surface. It can be observed that the photocatalytic activity increases with an increase in the number of adsorption abilities that are related to the specific surface and composition of the material.

### 3.7. Photo-Degradation Cycles

**Figure 15:**
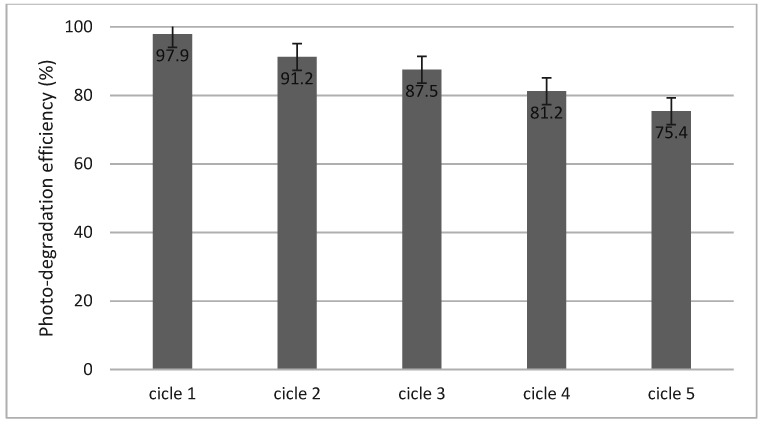
Cycling photo-degradation of cytarabine in the presence of BFO.

The material was tested in order to determine its stability over time. From the data obtained, it was found that the material has high stability and can be used for five photo-degradation cycles (data presented in Figure 15).

After all the cycles, the activity of the tested BFO material was lower than in the first cycle (decreased from 97.9% to 75.4%). The decrease in activity can be correlated with the decrease in the degree of adsorption. Unfortunately, the changes in the degree of adsorption were related to the mass loss of the BFO material (from 0.15 g to 0.08 g) during filtration. However, the results showed that the obtained photocatalyst possessed good long-term photocatalytic stability.

### 3.8. The Proposed Mechanism for the Photo-Degradation Process of Cytarabine

During the UV irradiation of the BFO composite, electron-hole pairs are successfully formed. Under irradiation, electrons from the valence band are transferred in the BFO material conduction band, where they can be freely transported along the material’s conductive network. Thus, the electron-hole pairs are well separated, and the recombination process is suppressed. In addition, a good separation of charge carriers leads to the improved production of reactive oxygen species, which greatly improves the photoactivity of BFO. Advanced oxidation processes have been successfully applied in cytarabine wastewater treatment [2].

The mechanism that occurs during the degradation of phenolic type materials was proposed below by De Heredia et al. [21]:

(11) $$H_{2}O_{2\;}+{\mathrm{Fe}}^{2+}\to {\mathrm{Fe}}^{3+}+{\mathrm{OH}}^{-}+{\mathrm{OH}}^{*}$$

(12) $$H_{2}O_{2\;}+{\mathrm{OH}}^{*}\to HO_{2}^{*}+H_{2}O$$

(13) $${\mathrm{OH}}^{*}+{\mathrm{Fe}}^{2+}\to {\mathrm{Fe}}^{3+}+{\mathrm{OH}}^{-}$$

(14) $${\mathrm{Fe}}^{3+}+H_{2}O_{2\;}\;\to {\mathrm{Fe}}^{2+}+H^{+}+HO_{2}^{*}$$

(15) $${\mathrm{Fe}}^{3+}+HO_{2}^{*}\;\to O_{2}+{{\mathrm{Fe}}^{2+}+H}^{+}$$

In the last step, the mineralization process takes place because of prolonged lighting and also as a result of the loss of active iron that becomes ferric hydroxide. The following reactions take place: [19]:

(16) $${\mathrm{Fe}}^{3+}+H_{2}O_{\;}\;\to {\mathrm{FeOH}}^{2+}+H^{+}$$

(17) $${\mathrm{FeOH}}^{2+}+H_{2}O_{\;}\;\to {\mathrm{Fe}(\mathrm{OH})}_{2}^{+}+H^{+}$$

(18) $${\mathrm{Fe}(\mathrm{OH})}_{2}^{+}+H_{2}O_{\;}\;\to {\mathrm{Fe}(\mathrm{OH})}_{3}+H^{+}$$

(19) $${\mathrm{Fe}(\mathrm{OH})}_{3}\to {\mathrm{Fe}(\mathrm{OH})}_{3}\;\mathrm{solid}$$

### 3.9. Evaluation of the Antimicrobial Effect of Cytarabine

The evaluation of the antimicrobial effect of cytarabine was performed by determining the lowest concentration (MIC) that killed 99.9% of microorganisms, established after incubation at 35 ± 2 °C for 24 h. The minimum bactericidal concentration (MBC) and minimum fungicidal concentration (MFC) were also established for the tested species. The results are presented in Table 6.

These results show that chemotherapy drugs commonly used in oncology, such as Kabi cytarabine, have an antimicrobial effect on strains of *E. coli* and *C. parapsilosis*. Similar results against *E. coli* have been observed in other studies [82,83].

Although the human normal flora contains a multitude of microbial species, including *E. coli* and *S. aureus* species, they can also be considered a pathogenic species that can produce opportunistic infections [84,85], especially in the case of patients with neoplasia, who have low immunity. The results obtained in the present study indicate that the chemotherapeutic agent, Kabi cytarabine, affects the growth of some microbes *(E. coli* and *Candida* sp.), so there is the possibility of producing an imbalance of the opportunistic bacterial flora and the possible favoring of the multiplication of multiresistant germs to the antibiotic, such as *P. aeruginosa* or *S. aureus* [51,86].

## 4. Conclusions

The aim of this work was to use a composite material based on iron oxide and bismuth for the photo-degradation of Kabi cytarabine, a very toxic cytostatic, present in aqueous solutions removed from the hospital system. The material was characterized by XRD, FT-IR, TG, AFM and SEM techniques. The size of the gap band was determined by UV spectroscopy and the specific surface was determined with the BET method.

In this work a new method used for cytarabine degradation was presented, using a photoactive semiconductor. The novelty of the obtained results originates from both creating new material as well as the successful degradation in the absence of additives that could increase the degradation speed.

The kinetics of the photo-degradation process was established. The material studied for the photo-degradation of cytarabine, BFO, according to the experiments carried out, has a remarkable photo-degradation efficiency of 97.9% for an initial cytarabine concentration of 10 mg/L, for the case of using 0.15 g of material, during 120 min of interaction with UV radiation at 3 cm from the irradiation source. The material resisted for five cycles of photo-degradation with good results.

Kabi cytarabine was found to be able to inhibit *E. coli* and *C. parapsilosis* strains in a dose-dependent manner. Gram-negative bacterial strains were more sensitive to exposure to cytarabine compared to Gram-positive ones. This study revealed that pyrimidine derivatives may be able to fight infections caused by *E. coli* and *C. parapsilosis*, which is very important in the medical world, where it has long been found that cancer patients often have difficult bacterial or fungal infections to fight.

## Figures and Tables

**Table 1:** Values obtained from AFM analysis.

Sample Name	Ironed Area (µm^2^)	Sa (µm)	Sq (µm)	Sp (µm)	Sv (µm)	Sy (µm)
BFO	972.815	0.388	0.485	0.880	−1.163	2.044

**Table 2:** Textural parameters of the synthesized material, BFO.

Surface Area, BET Method, m^2^/g	Pore Size Distribution, BJH Ads, nm	Pore size Distribution, BJH Ads, nm	Pore Width, DFT Ads, nm	Total Pore Volume, cm^3^/g	FHH Ads Neglecting Adsorbate Surface Tension Effects/Accounting for Adsorbate Surface Tension Effects, D
0.7 m^2^/g	3.857	3.354	4.125	0.002	1.7232/2.5744

**Table 3:** Determined values of irradiation [*I*]:

Distance between the UV Lamp and the Irradiated Sample [cm]	Irradiance [W m^−2^]
3	250
7	105
10	83

**Table 4:** Radiation doses.

Irradiation Time [s]	Irradiance [W·m^−2^]	Dosage [J m^−2^]
1800	250	450,000
3600	250	900,000
5400	250	1,350,000
7200	250	1,800,000

**Table 5:** Pseudo-first-order kinetic constant for cytarabine degradation.

Material	*K_a_* (min^−1^)	R^2^
Bi_x_Fe_1-x_O_y_	0.0307	0.9363

**Table 6:** MIC, MBC or MFC concentration.

Microbial Strains	MIC	MBC/MFC
*Staphylococcus aureus ATCC 25923*	2048 μg/mL	2084 μg/mL
*Escherichia coli ATCC 25922*	512 μg/mL	512 μg/mL
*Pseudomonas aeruginosa ATCC 27853*	2048 μg/mL	2048 μg/mL
*Candida parapsilosis ATCC 22019*	1024 μg/mL	1024μg/mL

## Data Availability

The data presented in this study are available on request from the corresponding author.
